# The paradox of AI content labeling: how clarity influences information avoidance via cognitive dissonance on social platforms

**DOI:** 10.3389/fpsyg.2026.1751670

**Published:** 2026-03-10

**Authors:** Zhixuan Gong, Danling Peng, Jinwei Cui, Zhuoru Lv

**Affiliations:** 1 School of Design, Hunan University, Changsha, Hunan, China; 2 School of Architecture and Art, Central South University, Changsha, Hunan, China; 3 College of Literature, Hunan Normal University, Changsha, Hunan, China; 4 Media and Arts Laboratory, School of Journalism and Cultural Communication, Zhongnan University of Economics and Law, Wuhan, Hubei, China

**Keywords:** AI-generated content, AI labels, cognitive dissonance, information avoidance, social media transparency, AI-mediated communication

## Abstract

**Introduction:**

The rapid growth of AI-generated content (AIGC) on social media has led to the introduction of AI disclosure labels to enhance transparency; however, emerging technologies such as Sora2 make it difficult for users to discern synthetic from human-created content, presenting challenges for both users and platform designers.

**Methods:**

This study investigates how different AI labels (clear, ambiguous, and no label) affect user behavior, focusing on information avoidance. We performed two online experiments (*N* = 760) to examine these effects in simulated social media scenarios (Bilibili and TikTok).

**Results:**

We found that ambiguous AI labels functioned as heuristic barriers that significantly increased information avoidance compared to clear or no labels. Cognitive dissonance was identified as a key mediator, where conflicting information led to discomfort and subsequent disengagement. Furthermore, factors such as label-content congruence and thematic relevance moderated these impacts.

**Discussion:**

These findings suggest that while AI disclosure labels are intended to improve transparency, ambiguous labels may inadvertently hinder user engagement, offering important implications for the design of transparency tools in AI-driven social media environments.

## 1 Introduction

Over the past decade, social media platforms have revolutionized content creation, enabling users to generate and share multimedia at unprecedented scales. The integration of generative artificial intelligence (AI) tools has accelerated this transformation, allowing for the rapid production of realistic images, videos, and text that often blur the distinction between human and machine authorship (Katsamakas and Sanchez-Cartas, 2024; Pan et al., 2024). This proliferation of AIGC has democratized creative expression but simultaneously introduced challenges related to authenticity. Users now encounter a hybrid ecosystem where synthetic media coexists with human-created content, raising concerns about deception and eroded credibility in digital interactions (Altay and Gilardi, 2024; NewsGuard, 2024).

Against this backdrop, the shift toward AI-augmented ecosystems presents significant hurdles for users navigating authenticity in algorithmically curated feeds. While user engagement is primarily driven by content relevance and intrinsic interest, dual-process theories suggest that in information-overloaded environments, individuals rely on heuristic cues to efficiently screen stimuli before deep processing (Chaiken et al., 1989; Sundar, 2020). We argue that in the specific context of generative AI, authenticity acts as a critical precondition for content utility, especially when truthfulness is expected. Implicitly, before a user invests cognitive resources to evaluate whether the content is personally interesting, they must first assess what the content is.

If a disclosure label signals unresolved uncertainty or potential deception, it may significantly diminish the content’s expected utility, potentially outweighing the initial appeal of the topic. Verifying the authenticity of unmarked material requires careful analysis, which is a demanding task that users typically avoid when they want to relax (Kahneman, 2011). Therefore, disclosure labels serve as essential indicators of credibility that help users quickly assess validity. Functioning as an upstream heuristic filter (Sundar, 2020), the label conditions the subsequent engagement likelihood, often preempting a deep evaluation of topical relevance. If it signals high processing costs or validity threats, it triggers a defensive avoidance response to preserve cognitive resources (Chaiken et al., 1989), or a defensive reaction to mitigate psychological discomfort (Golman et al., 2017). Thus, the signaling effect of the label acts as a conditional gatekeeper that can prompt avoidance even for potentially relevant content.

**Figure 1:**
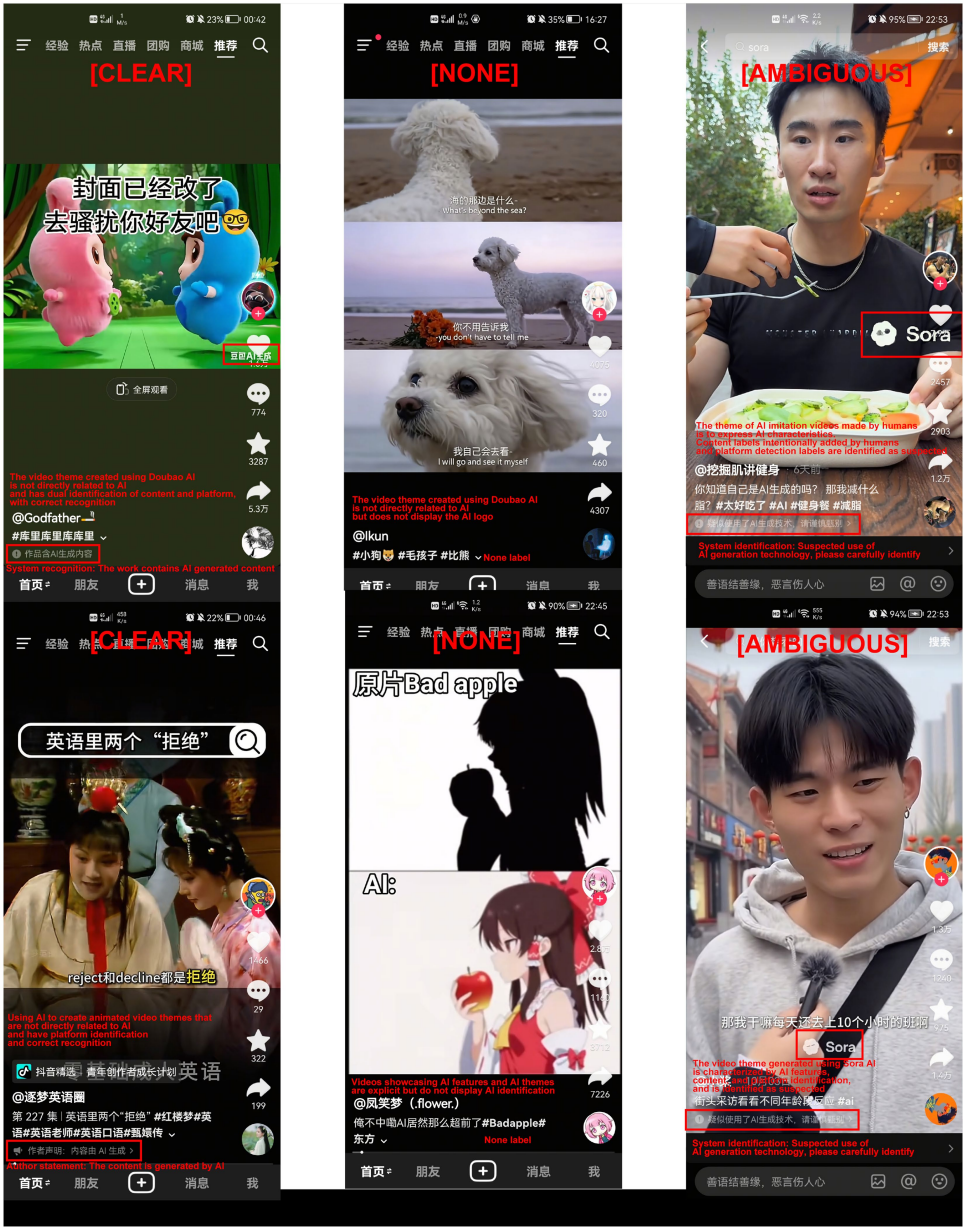
Examples of AI disclosure labels on Douyin used in the study. Source: The material obtained the video capture license from the creator of China TikTok platform.

To address these risks, platforms have introduced AI disclosure labels as key interventions. These labels, ranging from explicit declarations (e.g., content by AI) to ambiguous warnings (e.g., suspected AI-generated), aim to provide cues for trust calibration (Altay and Gilardi, 2024). However, unlike standardized moderation, existing label designs vary significantly in clarity. As shown in Figure 1, which provides an example of AI label usage on Douyin, these disclosures often lack consistency. Yet, the actual behavioral impact of these labels remains underexplored. While intended to clarify, they may paradoxically function as heuristic barriers that trigger defensive avoidance before users even evaluate the relevance of the content. Specifically, ambiguous labels that suggest but do not confirm AI involvement may generate unresolved uncertainty, prompting users to disengage rather than invest the cognitive effort to verify the content (Golman et al., 2017; Le et al., 2025).

This tension raises a critical theoretical gap: To what extent does label ambiguity impede the engagement process by disrupting the validity assessment? The core research question driving the study is: How does the type of AI label alter user avoidance of social media content, and by what psychological processes?

To address this, we propose that AI labels operate as a filtering mechanism where the perceived cost of verification undermines the expected gratification of consumption, moderated by label-content congruence and thematic relevance. We performed two online experiments (*N* = 760) to examine how different AI label types (none, clear, ambiguous) impact information avoidance behavior in simulated social media scenarios. We examined cognitive dissonance as a mediating factor and tested boundary conditions. These results advance understanding of cue processing in AI-augmented ecosystems and inform platform design by underscoring the risks of ambiguous disclosures (Liao and Wortman Vaughan, 2024).

## 2 Literature review

### 2.1 The rise of AI-generated content and the paradoxical role of disclosure labels in social media

The advent of generative AI tools has transformed digital content creation on social media since around 2023, enabling rapid production of realistic media and increasing the share of synthetic visuals (Corsi et al., 2024; European Union Agency for Law Enforcement Cooperation, 2024; NewsGuard, 2024). This proliferation raises concerns about authenticity, as users encounter media blurring human and machine creation, prompting regulatory responses like the European Union AI Act mandating labels for manipulated content (European Parliament and Council of the European Union, 2024; European Commission, 2025). TikTok and other platforms now use tags such as “Made with AI” to be more transparent, but the implementation of these tags is not consistent and may cause unintended user perceptions (TikTok, 2024; Pew Research Center, 2025a). Such marks are expected to improve transparency, but different circumstances cause different actions and unexpected opinions by the users.

Existing research on AI disclosure labels highlights their potential to support user awareness and limit misinformation, with clear labels helping distinguish AI from human content and calibrate trust (Epstein et al., 2023; Wittenberg et al., 2024; Wang et al., 2025). These findings align with HCI research on transparency cues strengthening accountability in algorithmic systems (Sundar, 2020; Molina and Sundar, 2022), and policy briefs emphasize ethical transparency even if persuasive effects are modest (Stanford HAI, 2025). However, disclosures are not uniformly beneficial; for instance, AI indications in prosocial advertising can lower evaluations through reduced authenticity (Baek et al., 2024; Qiu et al., 2025). Furthermore, an expanding body of evidence highlights paradoxical effects, where labels can erode trust, particularly in sensitive domains, leading to skepticism toward the information presented and reduced engagement (Altay and Gilardi, 2024; Schilke and Reimann, 2025; Walsh and Toff, 2025; Wang et al., 2025). Most empirical studies have focused on explicit labels, while the effects of more ambiguous warnings, which suggest potential AI involvement, may amplify uncertainty and confusion; yet, their everyday impacts remain underexplored (Wittenberg et al., 2024, 2025). Moreover, these studies predominantly rely on Western samples, potentially overlooking cultural differences in AI trust and label responses, especially among Asian users (Yam et al., 2023).

Even so, the body of work on AI labels is rather limited in scope. Most of the studies focus on the outcomes like perceived credibility, sharing intention or persuasiveness, and they neglect the defensive responses from users. According to the research of information avoidance, people sometimes avoid information that is unsettling or cognitively challenging to reduce the discomfort of anticipating this information or to maintain their motivated beliefs (Golman et al., 2017). In overstuffed social media feeds, ambiguous AI labels which cast doubt on authenticity can influence both what users believe and if they interact with the labeled material. But few study the comparison between explicit and ambiguous AI disclosures, or how labels might prompt disengagement and avoidance. This gap is addressed in the present study through concentrating on information avoidance as one of the outcomes of AI labeling.

### 2.2 Information avoidance as a key user response in digital media environments

Information avoidance constitutes a core psychological mechanism whereby individuals intentionally evade potentially beneficial information to safeguard emotional stability or mitigate distress (Sweeny et al., 2010; Golman et al., 2017). Drawing from decision theory and behavioral economics, this phenomenon arises not from apathy but from threats to beliefs, anxiety induction, or uncompensated cognitive demands (Golman et al., 2017). Sweeny et al. (2010) delineate a conceptual model of information avoidance, spanning awareness of information presence to deliberate rejection, modulated by anticipated regret and emotional burdens. Fundamentally, avoidance functions as a defense, enabling psychological balance amid dissonant inputs.

Within digital media, avoidance emerges through platform-specific actions leveraging features like endless feeds and algorithmic curation (Toff and Kalogeropoulos, 2020; Soroya et al., 2021). Users selectively mute terms, unfollow sources, or bypass content to sidestep unsettling topics, such as partisan news or health alerts (Soroya et al., 2021). In high-volume settings, this prioritizes emotional welfare over exhaustive intake, amplified by information overload leading to fatigue (Toff and Kalogeropoulos, 2020; Sheng et al., 2023).

However, existing literature tends to frame avoidance primarily as a reaction to content attributes, such as disinterest or affective threat involving fear of bad news (Sweeny et al., 2010; Golman et al., 2017).

Critically, these frameworks often implicitly assume that users evaluate the substance of the information before deciding to engage. We argue that in the era of generative AI, a distinct heuristic mechanism emerges where authenticity acts as a prerequisite for content utility. Drawing on the truth-default theory (Levine, 2014) and the framework of information utility (Golman et al., 2017), we posit that if content is perceived as potentially fabricated or deceptive, its informational and hedonic value is fundamentally compromised. In algorithmically curated feeds, users act as cognitive misers relying on heuristic cues to screen stimuli before engaging in systematic processing (Fiske and Taylor, 1984; Chaiken et al., 1989; Pennycook et al., 2020).

The presence of a suspected AI label creates ambiguity regarding the authenticity of the content and significantly lowers its perceived utility (Golman et al., 2017). This uncertainty often prompts users to disengage from the information, potentially overriding topical alignment. Consequently, the label acts as a preliminary screening mechanism where individuals choose avoidance as a quick strategy to bypass potentially invalid material (Chaiken et al., 1989; Sundar, 2020). This suggests that indicators of validity can effectively intercept the engagement process, functioning as a barrier independent of the user’s intrinsic interest in the subject (Pennycook et al., 2020).

Despite this, much of the scholarship on AIGC disclosures has focused on downstream outcomes such as trust erosion, credibility judgment, or persuasion (Altay and Gilardi, 2024; Wang et al., 2025), implicitly assuming that labels prompt users to engage in increased scrutiny. This perspective overlooks the exit option in an attention scarce ecosystem where rather than expending energy to scrutinize ambiguous content, users are more likely to simply scroll away. Current frameworks lack an explicit application of avoidance theory to the specific context of AI labeling, particularly regarding how the uncertainty of ambiguous labels might amplify this withdrawal. This study addresses this gap by positioning information avoidance as the dominant defensive response to ambiguous transparency cues, a process we explain through cognitive dissonance theory in the following sections.

### 2.3 Cognitive dissonance as the underlying mechanism linking AI labels to information avoidance

Cognitive dissonance theory, originally proposed by Festinger (1957), posits that individuals experience psychological discomfort when holding conflicting cognitions, such as incompatible beliefs, attitudes, or perceptions of behavior. This tension motivates efforts to reduce dissonance through attitude change, behavior modification, or selective information processing. A significant extension of this theory is the action-based model of dissonance (Harmon-Jones et al., 2015), which emphasizes that dissonance arises particularly when conflicting cognitions impede effective action or decision-making, prompting a sense of urgency to resolve the inconsistency for adaptive functioning. While heuristic models explain cue processing in high-load environments (Chaiken et al., 1989), cognitive dissonance theory uniquely captures the affective tension that arises when contradictory signals block the user’s ability to form a coherent interpretation of reality.

Applied to the context of AIGC disclosures, we argue that cognitive dissonance arises not merely from the cognitive effort required for processing, but from an epistemic conflict between the user’s default expectation of authenticity and the uncertainty introduced by the label. Social media users typically operate under a truth-default state (Levine, 2014), engaging with content intuitively and assuming it to be a valid representation of reality unless proven otherwise (Pennycook et al., 2020). However, the appearance of an AI disclosure label, particularly an ambiguous one, disrupts this coherence.

Specifically, we propose that ambiguous AI labels trigger dissonance by creating a state of unresolved verification. When a user encounters realistic content with a label indicating potential synthetic origin, two contradictory cognitions collide: the visual evidence suggesting the content is authentic and hedonic, versus the platform’s warning implying it may be synthetic or invalid. This conflict creates psychological discomfort stemming from the inability to reconcile these opposing cues (Bode and Vraga, 2015; Metzger et al., 2020). Unlike clear labels which offer a definitive categorization allowing immediate belief updating, ambiguous labels maintain a state of epistemic uncertainty. The user is caught between the desire to consume the content and the suspicion raised by the label. According to the action-based model, this state is highly aversive because it blocks the user’s primary goal of effortless consumption, creating a tension that demands resolution (Harmon-Jones et al., 2015).

Therefore, avoidance serves as a highly efficient dissonance reduction strategy (Sweeny et al., 2010; Golman et al., 2017). To resolve this psychological discomfort and restore cognitive consistency without engaging in a lengthy and uncertain verification process, users strategically choose to disengage from the stimulus entirely. By scrolling away, the user removes the source of the conflicting cognitions, thereby rapidly restoring psychological consistency. This mechanism explains the paradoxical behavioral outcome where transparency interventions drive users away from potentially relevant information, as the immediate relief from epistemic tension takes precedence over the potential utility of the content.

**Figure 2:**
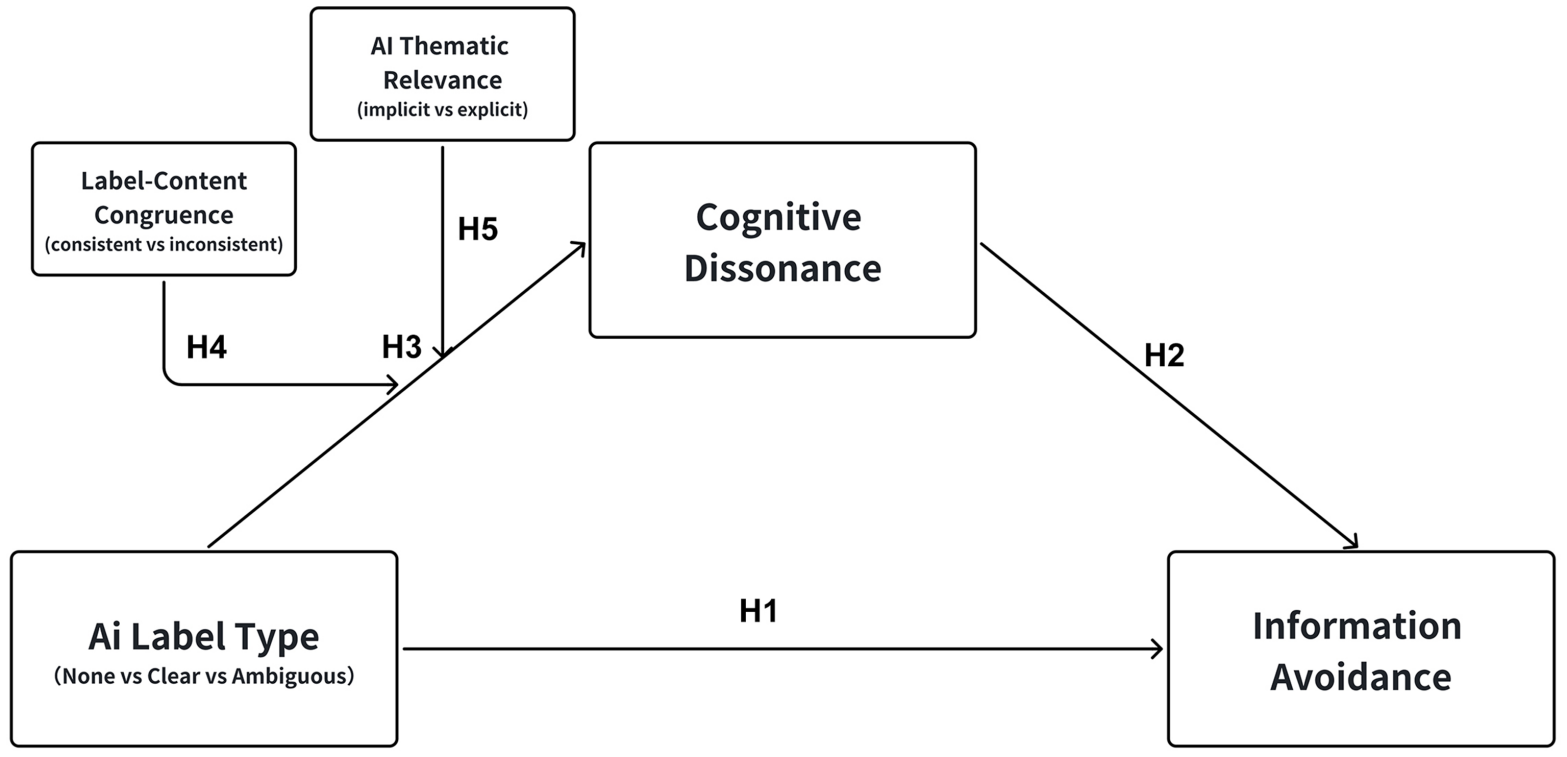
The conceptual model of the study. Source: Author’s own work.

Based on this logic, we hypothesize a conceptual model (see Figure 2) in which the type of AI label influences cognitive dissonance, defined as the psychological tension arising from conflicting authenticity cues, which in turn drives information avoidance.

*H1*: AI label display on social media will affect users’ information avoidance.

*H1a*: When the displayed label is clear, user avoidance will increase compared to no label.

*H1b*: When the displayed label is ambiguous, user avoidance will increase compared to no label (and more than clear labels).

*H2*: Cognitive dissonance will positively predict information avoidance.

*H3*: Cognitive dissonance mediates the relationship between label type and information avoidance.

By distinguishing label clarity levels and establishing dissonance as the mediating mechanism, this research provides empirical evidence regarding why AI disclosures, particularly ambiguous ones, may paradoxically heighten disengagement. This extends cognitive dissonance applications into AIGC transparency debates and sets the stage for examining boundary conditions in the next section.

### 2.4 Boundary conditions: the moderating roles of label-content congruence and AI thematic relevance

While the core model discussed earlier elucidates how cognitive dissonance mediates the impact of AI label type on information avoidance, this relationship is likely contingent on contextual factors that amplify or attenuate dissonance arousal. Social media content varies widely in its alignment with labels and thematic focus, necessitating the exploration of boundary conditions to enhance theoretical precision and practical relevance.

Grounded in cue congruency effects derived from the heuristic-systematic model (Chaiken et al., 1989; Einwiller and Bohner, 2024) and motivated reasoning frameworks (Kunda, 1990; Taber and Lodge, 2006), we propose two moderators, label-content congruence and AI thematic relevance, that influence the path from label type to cognitive dissonance. This yields a moderated mediation model (illustrated in Figure 2), where the indirect effect of labels on avoidance through dissonance strengthens under conditions of mismatch or unexpectedness, explicitly linking to cue utilization theories by showing how inconsistent cues disrupt processing fluency in HCI contexts (Sundar, 2008; Shin, 2020). By incorporating these moderators, the study offers an integrated analysis of situational contingencies in AIGC disclosure effects, addressing gaps in prior work that assumes thematic uniformity or ignores non-Western user attitudes toward AI ethics (Dang and Li, 2025; Pew Research Center, 2025b).

Label-content congruence denotes the match between the platform’s label and the actual content generation method, dichotomized as consistent (accurate labeling, e.g., AI content correctly tagged as such) or inconsistent (mismatch, e.g., human-created video erroneously labeled as AI). Cue consistency theory asserts that aligned cues promote processing fluency and reduce conflict, whereas discrepancies heighten vigilance and negative affect by signaling unreliability (Chaiken et al., 1989; Sundar, 2008).

In HCI contexts, lower perceived fairness and transparency of algorithmic systems exacerbate user frustration and disengagement, as they undermine trust in the system (Shin, 2020). For AIGC labels, incongruence intensifies dissonance because it adds a layer of perceived deception or error to the authorship cue: users question not only the content’s origin but also the platform’s competence. Users may generalize their skepticism beyond the targeted content, as prominent interventions can reduce misperceptions but simultaneously increase skepticism toward accurate information (Hoes et al., 2024).

Consistent conditions, in contrast, facilitate resolution, mitigating the dissonance spike. Prior research on misinformation flags supports this, showing that warning labels can have unintended spillover effects, such as increasing skepticism toward unlabeled or even accurate content (Pennycook et al., 2020; Martel and Rand, 2023; Hoes et al., 2024). However, no studies have tested congruence as a moderator in multi-level label clarity designs, a gap this work addresses to reveal why real-world labeling errors might disproportionately harm engagement.

*H4*: Label-content congruence moderates the effect of label type on cognitive dissonance, such that the positive impact of labels is stronger under inconsistent conditions (vs. consistent).

AI thematic relevance refers to the explicitness of AI-related elements in the video’s topic, categorized as explicit (overt AI focus, e.g., demonstrations of Sora 2 capabilities) or implicit (no direct AI mention, e.g., general entertainment clips). Motivated reasoning theory suggests that information processing is fundamentally directed by individuals’ goals and motivations (Kunda, 1990). In contexts with implicit AI themes (e.g., entertainment), users typically operate in an intuitive mode characterized by low cognitive reflection rather than a drive for accuracy (Pennycook et al., 2020). Consequently, the intrusion of an ambiguous label in such low-stakes settings acts as a jarring disruption to the hedonic experience. This mismatch triggers an automatic negative affective response and “motivated skepticism” toward the disturbing cue (Taber and Lodge, 2006), thereby rendering the unexpected label more dissonant and prompting withdrawal.

Complementing expectancy violation theory, implicit themes create a greater shock when labels intrude, as users lack priming for AI skepticism and experience the cue as an unwarranted intrusion on neutral content (Burgoon, 2015). In communication studies, labels on non-thematic material (e.g., lifestyle deepfakes) can elicit reactance and withdrawal, as authenticity expectations are violated (Eslami et al., 2019; Vaccari and Chadwick, 2020).

For AIGC, explicit themes buffer effects by aligning with user expectations (“This is about AI, so labels make sense”), while implicit ones escalate dissonance, particularly for ambiguous labels that demand unmotivated verification. This moderator has been underexplored within labeling literature that usually assumes thematic uniformity (Epstein et al., 2023) without considering the fact that a large part of AI-related exposure takes place in implicitly themed contexts instead of clearly AI-focused content (Pew Research Center, 2025a), which may make unexpected AIGC labels particularly disruptive in diverse cultural settings (Dang and Li, 2025).

*H5*: AI thematic relevance moderates the effect of label type on cognitive dissonance, such that the positive impact of labels is stronger for implicit themes (vs. explicit).

In sum, this moderated mediation framework synthesizes dissonance theory with contextual cue processing, uncovering when transparency initiatives falter. Innovations include (1) empirically validating ambiguous labels as a distinct, more potent trigger than clear ones; (2) introducing dual moderators to explain variability in prior inconsistent findings (e.g., why labels backfire selectively in mismatched or non-AI content); and (3) testing the model in simulated social media scenarios for ecological validity.

These advances extend AIGC and avoidance literatures, guiding platforms to refine labeling strategies and avoid counterproductive outcomes. The ensuing sections detail two experimental studies evaluating these hypotheses.

## 3 Overview of study

The present research tests the proposed moderated mediation model (Figure 2) across two experimental studies with a constructive replication design that progresses from internal to ecological validity. Study 1 (*N* = 371) establishes the baseline effects in a controlled setting by testing the main effect of AI label type (no label vs. clear vs. ambiguous) on information avoidance (H1–H1b), the positive role of cognitive dissonance (H2), and its mediating function (H3). In order to eliminate potential confounding effects from the proposed moderators, all stimuli in Study 1 consistently featured explicit AI themes and exhibited perfect congruence between labels and content. This controlled experimental design supports inferences regarding the causal pathways from label type to cognitive dissonance and subsequently to information avoidance.

Study 2 (*N* = 389) replicates these effects while introducing the two moderators in a full 3 × 2 × 2 between-subjects design, thereby testing the moderated mediation hypotheses (H4–H5) in a more realistic TikTok-like environment with varied across both congruence (consistent vs. inconsistent) and thematic relevance (explicit vs. implicit). This sequential research approach first confirms the primary psychological process under conservative, tightly restricted circumstances. It then explores the boundary conditions of this mechanism within a more naturalistic digital context, thereby offering cumulative and robust empirical support for understanding when and why AI disclosure labels, particularly those categorized as ambiguous, may paradoxically lead to increased user disengagement.

The study was conducted in accordance with the Declaration of Helsinki and was approved by the ethical review board of the corresponding author’s institution.

## 4 Study 1

Study 1 tested the core hypotheses of the proposed model in a relatively controlled media context: whether the presence of an AI label affects information avoidance (H1) and, more specifically, the directional effects of clear versus ambiguous labels (H1a–H1b); whether cognitive dissonance positively predicts information avoidance (H2); and whether cognitive dissonance mediates the relationship between AI label type and information avoidance (H3). To ensure internal validity for causal inference and to rule out the influence of the moderators that are explicitly examined in Study 2, Study 1 adopted an explicit AI-related theme in the stimulus materials and strictly aligned information sources with labels, such that the platform label always matched the actual mode of content generation in the video.

### 4.1 Method

Participants were recruited online via the Credamo platform (*N* = 391) and received monetary compensation for their participation. Twenty participants were excluded because they failed the attention check or had not used relevant social media platforms for an extended period, resulting in a final valid sample of 371 participants. Demographic characteristics are shown in Table 1. In terms of gender, 43.7% identified as male (*n* = 162) and 56.3% as female (*n* = 209). The mean age was 28.86 years (SD = 9.840). With respect to educational background, participants with a bachelor’s degree constituted the largest group (54.4%, *n* = 202), followed by those with an associate degree (25.6%, *n* = 95), high school or below (14.3%, *n* = 53), and a master’s degree or above (5.7%, *n* = 21). Regarding daily internet use, 4.3% reported less than 1 h per day (*n* = 16), 65.5% reported 1–5 h (*n* = 243), 24.8% reported 5–10 h (*n* = 92), and 5.4% reported more than 10 h (*n* = 20).

**Table 1:** Demographic characteristics of participants in studies 1 and 2.

Demographic characteristics	Study 1	Study 2
Total sample	371	389
Male	43.7% (162)	44.50% (174)
Female	56.3% (209)	55.50% (215)
Mean age	28.86	32.15
Age SD	9.84	10.28
Education (bachelor’s)	54.4% (202)	46.5% (180)
Education (associate)	25.6% (95)	30.3% (118)
Education (high school or below)	14.3% (53)	15.2% (59)
Education (master’s or above)	5.7% (21)	7.2% (28)
Internet use (<1 h)	4.3% (16)	4.6% (18)
Internet use (1–5 h)	65.5% (243)	63.5% (247)
Internet use (5–10 h)	24.8% (92)	26% (101)
Internet use (>10 h)	5.4% (20)	5.9% (23)

Study 1 employed a single-factor between-subjects experimental design. The dependent variable was information avoidance, and the sole independent variable was AI label type, with three levels: no label, clear label, and ambiguous label. The 371 participants were randomly assigned to one of the three label conditions, and each participant viewed three short videos. The experimental scenario simulated the viewing environment of a video-based social media platform represented by Bilibili.

Participants viewed 15–30 s short videos featuring explicit AI themes (e.g., “How to Create Ultra-Realistic Videos with Sora 2”). A pretest (*N* = 60) validated these stimuli, confirming high AI thematic relevance (M = 6.07, SD = 1.01) and strong consistency between the content and its generation method (M = 6.10, SD = 0.90). All videos were posted by an account with the same neutral avatar and a virtual nickname to simulate the standardized labeling format commonly used by the platform as of June 2025. The identification label was overlaid in grey, 12-point font beneath the video title. In the no-label condition, this area was left blank. In the clear-label condition, it read “Content generated by AI,” whereas in the ambiguous-label condition, it read “Suspected AI, please verify.” After reading the online informed consent form, participants proceeded to the experiment. Immediately after viewing the videos, they completed scales measuring cognitive dissonance, information avoidance, and other variables. Finally, participants reported their demographic information before being debriefed.

As the dependent variable, information avoidance was measured with three items adapted from previous research (Dai et al., 2020). To align with the experimental context, we employed a contextual adaptation strategy, modifying the original items (which focused on WeChat interactions) to refer to algorithmically recommended content on social media. The scale showed good internal consistency (*α* = 0.851). A sample item is, “I intend to avoid information from some sources or videos like this on social media.” Serving as the mediator, cognitive dissonance was measured with three items adapted from existing measures (Metzger et al., 2020; Song et al., 2021). We modified the target object of the original items, which originally assessed discomfort regarding controversial topics, to specifically focus on the authenticity of the video content and labels. The scale demonstrated high internal consistency (*α* = 0.879). A sample item is, “I felt confused while reading/viewing this video story” (see Table 2 for the full list of items).

**Table 2:** Measurement items, reliability, and sources.

Construct & items	Cronbach’s *α* (Study 1/Study 2)	Source
Information avoidance	0.851/0.827	Adapted from Dai et al. (2020)
I will accept information selectively related to this type of content on social media		
I will refuse to accept some similar content on social media		
I intend to avoid information from some sources or videos like this on social media		
Cognitive dissonance	0.879/0.891	Adapted from Metzger et al. (2020) and Song et al. (2021)
The information source or label on this video makes me uncomfortable		
I felt confused while reading/viewing this video story		
This video story made me question my own beliefs about content authenticity		
Source consistency	0.820/0.835	Li and Yang (2024)
This source seems very professional		
This source is very trustworthy		
This source’s profile (avatar/name) is very attractive		

Multiple manipulation checks were used to assess the effectiveness of the experimental manipulations. First, perceived certainty about the production tool was measured with the item, “Based on the media presentation, how certain are you about the tool used to produce the video content?” (1 = very uncertain, 7 = very certain), to capture participants’ perception of AI label type. Second, source consistency was assessed with three items from Li and Yang (2024) (e.g., “This source seems very professional”), yielding an internal consistency of *α* = 0.820. Third, label–content congruence was measured with the item, “To what extent do you agree that the platform label (including whether a label is present and what it says) matches the video’s actual mode of generation (AI-generated vs. human-made)?” (1 = strongly disagree, 7 = strongly agree). Fourth, AI thematic relevance was measured with the item, “To what extent is the theme of this video related to AI?” (1 = not at all related, 7 = very highly related). All items were rated on 7-point Likert scales.

An attention check item (“please select 3”) was placed in the questionnaire to further improve data quality. It took about 10 min to finish the experiment. At the end, they were given a short debrief on what kind of content was created by an AI and how AI generated content is verified by platforms.

Data were analyzed using SPSS 28.0. To test H1 (the main effect of AI label type on information avoidance), a one way ANCOVA was performed, with AI label type as the fixed factor, information avoidance as the dependent variable, and age, gender, education level, and daily internet use as covariates. *Post-hoc* multiple comparisons were done with the LSD procedure, and Cohen’s *d* is used to express the effect size. We interpreted the magnitude of these effects based on Cohen’s benchmarks, where *d* ≈ 0.2 indicates a small effect, *d* ≈ 0.5 a medium effect, and *d* ≈ 0.8 a large effect (Cohen, 1992). To test H2 and H3, we use PROCESS Model 4 (Hayes, 2022), with 5,000 bootstraps resamples to get the indirect effects and the 95% confidence intervals.

### 4.2 Result

Before testing the hypotheses, we examined the distributional characteristics of the key continuous variables. Table 3 presents the descriptive statistics and normality diagnostics for the key variables. Skewness and kurtosis values for all variables fell well within the recommended range of ±1.5 (George and Mallery, 2019), confirming that the data satisfy the assumption of normality required for subsequent analyses.

**Table 3:** Descriptive statistics and normality diagnostics for key variables (pooled sample).

Study	Variable	Mean	SD	Skewness	Kurtosis
Study 1	Information avoidance	3.69	1.28	0.173	−0.709
	Cognitive dissonance	4.78	1.2	−0.406	−0.602
	Source consistency	4.25	1.07	−0.259	0.621
Study 2	Information avoidance	3.66	1.47	0.219	−0.452
	Cognitive dissonance	4.47	1.59	−0.153	−0.734
	Source consistency	4.26	1.12	−0.393	−0.71

#### 4.2.1 Manipulation checks

The manipulation check indicated that the AI label manipulation was successful. Participants’ ratings of certainty about the production tool differed significantly across the three label conditions: no-label condition (M = 1.98, SD = 0.82), clear-label condition (M = 5.60, SD = 1.17), and ambiguous-label condition (M = 3.76, SD = 1.35), *F*(2, 368) = 329.34, *p* < 0.001. Participants could clearly distinguish among the different labeling conditions.

For source consistency, there were no significant differences across conditions: no label (M = 4.20, SD = 0.99), clear label (M = 4.24, SD = 1.01), ambiguous label (M = 4.33, SD = 1.22), *F*(2, 368) = 0.450, *p* = 0.638, indicating that participants’ evaluations of source professionalism and reliability were generally comparable across label conditions.

Similarly, label–content congruence did not differ significantly across conditions: no label (M = 6.02, SD = 1.18), clear label (M = 6.24, SD = 0.91), ambiguous label (M = 6.14, SD = 1.00), *F*(2, 368) = 1.471, *p* = 0.231. Thus, under all three label types, participants generally perceived a high degree of match between labels and the actual mode of content generation.

No significant differences were found in perceived AI thematic relevance across conditions: no label (M = 5.81, SD = 1.15), clear label (M = 5.86, SD = 1.12), ambiguous label (M = 5.93, SD = 1.12), *F*(2, 368) = 0.331, *p* = 0.718. Taken together, these results indicate that Study 1 successfully manipulated AI label type while maintaining comparable levels of source consistency, label–content congruence, and an explicit AI-related theme across conditions.

#### 4.2.2 Information avoidance

**Figure 3:**
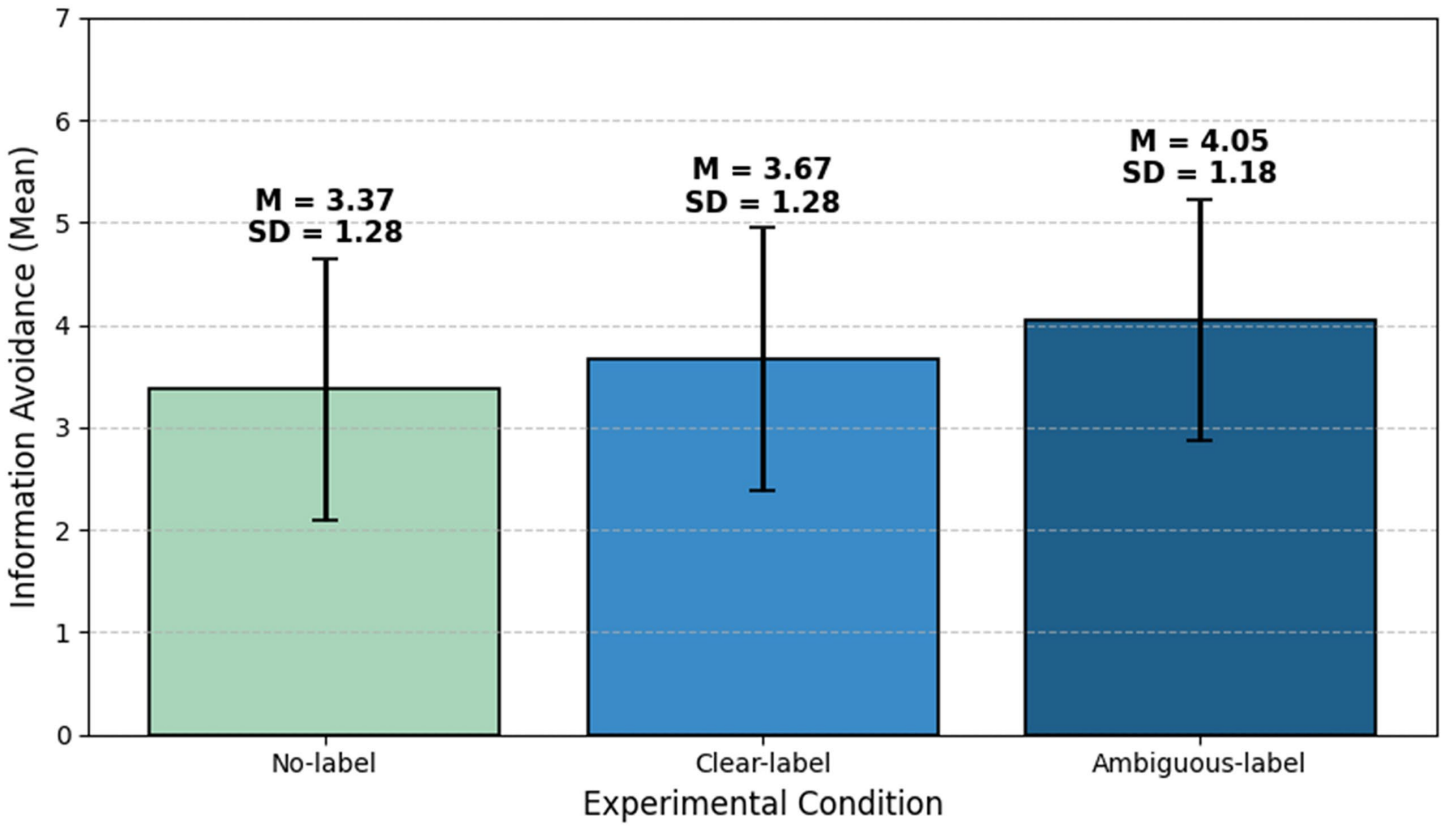
Mean information avoidance scores by label type in Study 1. Source: Author’s own work.

Regarding information avoidance, the ANCOVA revealed a significant main effect of AI label type, *F*(2, 364) = 9.88, *p* < 0.001, *η*^2^ = 0.051. *Post-hoc* comparisons showed that, after controlling for demographic covariates, information avoidance was highest in the ambiguous-label condition (M = 4.05, SD = 1.18). Avoidance in this condition was significantly higher than in the no-label condition (M = 3.37, SD = 1.28), *t*(364) = 4.44, *p* < 0.001, Cohen’s *d* = 0.57, and also higher than in the clear-label condition (M = 3.67, SD = 1.28), *t*(364) = 2.49, *p* = 0.040, Cohen’s *d* = 0.32. By contrast, the difference between the clear-label and no-label conditions was not significant, *t*(364) = 1.99, *p* = 0.141, Cohen’s *d* = 0.25. Figure 3 displays the mean information avoidance scores for the three AI label conditions in Study 1. None of the covariates exerted a significant effect. Overall, H1 was supported: ambiguous labels significantly increased information avoidance relative to no labels, supporting H1b, whereas the effect of clear labels relative to no labels was non-significant, and H1a was not supported.

#### 4.2.3 Mediation analysis

The mediation analysis further supported H2 and H3. Regression results indicated that cognitive dissonance positively predicted information avoidance, *β* = 0.62, *t* = 13.66, *p* < 0.001: higher levels of cognitive dissonance were associated with greater tendencies to avoid information, consistent with H2. Using PROCESS Model 4 to examine the indirect effect of AI label type on information avoidance via cognitive dissonance, we found that the indirect effect of clear labels (vs. no label) was 0.20 [SE = 0.10, 95% CI (0.01, 0.40)], and the indirect effect of ambiguous labels (vs. no label) was 0.44 [SE = 0.10, 95% CI (0.25, 0.63)], with the latter being stronger.

Regarding direct effects, after controlling for cognitive dissonance, the direct effect of clear labels (vs. no label) on information avoidance was not significant, *β* = 0.11, *p* = 0.386, whereas the direct effect of ambiguous labels (vs. no label) remained significant, *β* = 0.27, *p* = 0.042. This pattern suggests that the effect of clear labels on information avoidance was fully mediated by cognitive dissonance, whereas the effect of ambiguous labels was only partially mediated. Overall, cognitive dissonance played an important mediating role between AI label type and information avoidance, supporting H3.

### 4.3 Discussion

The findings of Study 1 supported H1, H1b, H2, and H3, whereas H1a was not supported. These results are consistent with previous findings on defensive mechanisms underlying information avoidance (Dai et al., 2020), as well as research on cognitive dissonance as a model of psychological discomfort (Metzger et al., 2020; Song et al., 2021). They underscore that ambiguous labels induce avoidance by amplifying uncertainty. Building on these findings, Study 2 incorporates label–content congruence and AI thematic relevance as moderators to further examine the boundary conditions of these effects.

## 5 Study 2

Study 2 aimed to replicate the core effect of AI labels on information avoidance observed in Study 1 in the context of short-video social media platforms represented by TikTok (Douyin), and to further examine the mediating role of cognitive dissonance in the relationship between label type and information avoidance (H3). Beyond this, Study 2 explicitly introduced two contextual variables:label–content congruence (whether the label matches the video’s actual generation method; H4) and AI thematic relevance (explicit vs. implicit AI-related theme; H5)—to construct and test a moderated mediation model and thus investigate the boundary conditions under which AI label type influences information avoidance via cognitive dissonance across different media contexts. Compared to the Bilibili-based simulation in Study 1, Study 2 shifted to a TikTok-based scenario to capture a more dynamic, algorithm-driven short-video viewing environment.

### 5.1 Method

Participants were randomly recruited via the Credamo platform (*N* = 415) and received monetary compensation, consistent with Study 1. Twenty-six participants were excluded for failing the attention check or extended non-use of relevant platforms, yielding a final sample of 389. Among them, 55.5% were female, with an average age of 32.15 years (SD = 10.28). The participants’ occupations and education levels are presented in Table 1. Educational background: Those who obtained a bachelor’s degree made up the largest proportion (46.5%, *n* = 180), followed by an associate degree (30.3%, *n* = 118), high school or lower (15.2%, *n* = 62), and master’s degree or higher (7.2%, *n* = 29). Daily internet use: 4.6% < 1 h (*n* = 18), 63.5% 1–5 h (*n* = 247), 26% 5–10 h (*n* = 101), 5.9% > 10 h (*n* = 23).

For sample size estimation, Study 2 conducted an *a priori* power analysis using G*Power 3.1 (Faul et al., 2007) for a three-way interaction in a 3 × 2 × 2 factorial design. Assuming a medium effect size of *f* = 0.25 (*η*^2^ = 0.06), statistical power of 0.80, significance level *α* = 0.05, numerator degrees of freedom = 2, and 12 groups, the analysis indicated that at least 158 participants were required to detect the three-way interaction. The final sample size of *N* = 389 far exceeded this minimum, ensuring robust statistical power and high sensitivity for testing the hypothesized moderated mediation effects.

**Figure 4:**
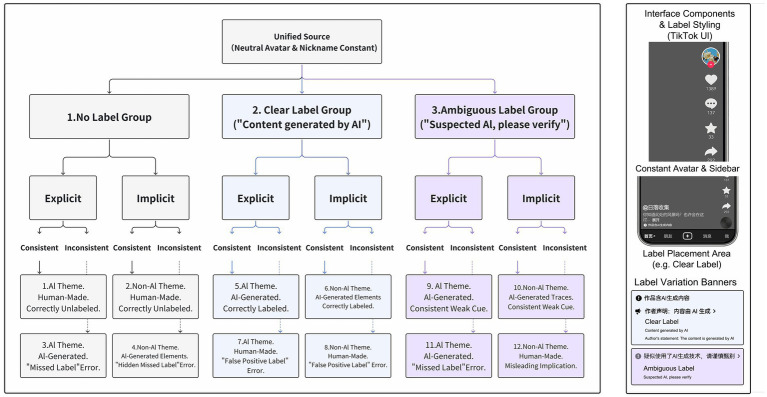
Experimental design and stimulus manipulation logic for Study 2. Source: Author’s own work.

Study 2 adopted a 3 (AI label type: no label vs. clear label vs. ambiguous label) × 2 (label–content congruence: consistent vs. inconsistent) × 2 (AI thematic relevance: explicit vs. implicit) between-subjects experimental design, resulting in 12 experimental conditions. The 389 participants were randomly assigned to one of these conditions, and each participant viewed three short videos. The experimental context simulated a typical viewing scenario on TikTok (Douyin), a social media platform centered on short-video content (see Figure 4 for an overview of the experimental design and stimulus manipulation logic).

With respect to stimulus materials, Study 2 manipulated AI thematic relevance (explicit vs. implicit) and label–content congruence (whether the label text matched the video’s actual mode of generation) at the video level. As in Study 1, all videos were posted by an account with the same neutral avatar and virtual nickname to maintain consistency at the source level. The presentation of platform labels followed common practices on the platform as of June 2025, appearing as text overlaid beneath the video title: in the no-label condition, this area was blank; in the clear-label condition, it read “Content generated by AI”; and in the ambiguous-label condition, it read “Suspected AI, please verify.” Unlike Study 1, Study 2 intentionally introduced mismatches between labels and actual generation methods in some conditions and embedded AI-related themes implicitly in others to reflect more complex and diverse real-world platform scenarios.

Measurement instruments in Study 2 were consistent with those used in Study 1. Information avoidance was assessed with three items adapted from the literature, yielding an internal consistency of *α* = 0.827 (Dai et al., 2020). Cognitive dissonance was measured using three items adapted from prior studies (Metzger et al., 2020; Song et al., 2021), with an internal consistency of *α* = 0.891 (see Table 2 for the full list of items).

Manipulation checks focused on four dimensions. First, AI label type was assessed by asking participants to rate “Based on the label, how certain are you about the tool used to produce the video content?” (1 = very uncertain, 7 = very certain), to test whether participants perceived the label manipulation. Second, source consistency was measured with the same three items as in Study 1 (e.g., “This source seems very professional.”), with an internal consistency of *α* = 0.835. Third, label–content congruence was measured with the item, “To what extent do you agree that the platform label matches the actual content of the video?” (1 = strongly disagree, 7 = strongly agree), to verify the effectiveness of the consistency versus inconsistency manipulation. Fourth, AI thematic relevance was measured with the item, “To what extent is the video’s theme related to AI?” (1 = not at all related, 7 = very highly related), to assess the explicit versus implicit AI topic manipulation. After completing the main tasks, participants reported demographic information. As in Study 1, an attention check item was embedded to ensure data quality.

Regarding data analysis, Study 2 first examined the effectiveness of all manipulations. Subsequently, three-way ANOVAs were conducted with information avoidance and cognitive dissonance as dependent variables, respectively, using AI label type, label–content congruence, and AI thematic relevance as independent variables, and gender, age, education level, and daily internet use as covariates. Mediation analyses employed PROCESS Model 4 (Hayes, 2022), with AI label type as the independent variable, cognitive dissonance as the mediator, and information avoidance as the dependent variable, to test the mediating role of cognitive dissonance. Further moderated mediation analyses used PROCESS Model 9 (Hayes, 2022), with label–content congruence and AI thematic relevance as dual moderators on the path from the independent variable to the mediator. Conditional indirect effects and their bias-corrected confidence intervals were estimated using 5,000 bootstrap samples.

### 5.2 Result

#### 5.2.1 Manipulation checks

For the AI label manipulation, participants’ ratings of certainty about the production tool differed significantly across the three label conditions, indicating that they could distinguish between the no-label, clear-label, and ambiguous-label conditions: no label (M = 2.12, SD = 1.00), clear label (M = 5.67, SD = 1.15), ambiguous label (M = 3.63, SD = 1.28), *F*(2, 386) = 302.070, *p* < 0.001.

For source consistency, differences across label conditions were not significant: no label (M = 4.18, SD = 1.14), clear label (M = 4.26, SD = 1.12), ambiguous label (M = 4.32, SD = 1.11), *F*(2, 386) = 0.476, *p* = 0.621, suggesting that AI label type did not systematically alter participants’ overall evaluations of source professionalism and credibility.

For label–content congruence, participants in the inconsistency condition perceived significantly lower consistency than those in the consistency condition: inconsistent label condition (M = 3.62, SD = 1.546) versus consistent label condition (M = 6.19, SD = 0.968), *t*(387) = 19.261, *p* < 0.001.

For AI thematic relevance, participants in the implicit topic condition perceived significantly lower AI-relatedness than those in the explicit topic condition: implicit AI topic (M = 3.31, SD = 1.540) versus explicit AI topic (M = 5.84, SD = 1.165), *t*(387) = 18.216, *p* < 0.001. Together, these results demonstrate successful manipulations of AI label type, label–content congruence, and AI thematic relevance in Study 2.

#### 5.2.2 Information avoidance

**Figure 5:**
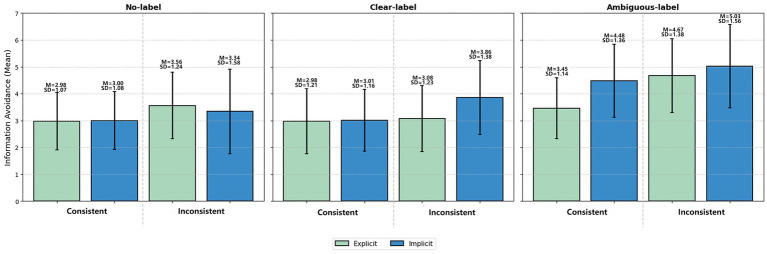
Mean information avoidance scores across experimental conditions in Study 2. Source: Author’s own work.

A 3 × 2 × 2 ANOVA was conducted on information avoidance, controlling for gender, age, education level, and daily internet use. The main effect of AI label type was significant, *F*(2, 373) = 35.42, *p* < 0.001, $\eta_{p}^{2}$ = 0.160 (large effect). Detailed descriptive statistics (M and SD) for all conditions are presented in Figure 5. Bonferroni-corrected *post hoc* comparisons confirmed that ambiguous labels elicited significantly higher information avoidance compared to both the no-label condition (Cohen’s *d* = 0.88, large effect) and the clear-label condition (Cohen’s *d* = 0.85, large effect; both *p* < 0.001). In contrast, clear labels did not differ significantly from the no-label baseline (*p* = 0.524).

The main effect of label–content congruence was also significant, *F*(1, 373) = 21.27, *p* < 0.001, $\eta_{p}^{2}$ = 0.054 (medium effect). Information avoidance was significantly higher in the inconsistency condition (M = 3.96, SD = 1.56) than in the consistency condition (M = 3.31, SD = 1.28). The main effect of AI thematic relevance was likewise significant, *F*(1, 373) = 6.59, *p* = 0.011, $\eta_{p}^{2}$ = 0.017 (small effect), with participants in the implicit topic condition (M = 3.81, SD = 1.56) showing higher information avoidance than those in the explicit topic condition (M = 3.50, SD = 1.35). Figure 5 displays the mean information avoidance scores for all 12 experimental conditions in Study 2.

No significant two-way or three-way interactions were observed for information avoidance, with all *p*-values exceeding 0.067. The effects of all covariates were also non-significant (all *p*-values > 0.071). Overall, in a TikTok-style short-video social media context, Study 2 replicated the core pattern observed in Study 1: ambiguous labels significantly increased information avoidance relative to both no labels and clear labels, whereas the difference between clear and no labels remained non-significant.

#### 5.2.3 Cognitive dissonance

A 3 × 2 × 2 ANOVA was also conducted on cognitive dissonance, controlling for the same covariates. The main effect of AI label type was significant, *F*(2, 373) = 48.54, *p* < 0.001, $\eta_{p}^{2}$ = 0.207 (large effect). Bonferroni-corrected post hoc tests showed that clear labels (M = 4.16, SD = 1.39) elicited significantly higher cognitive dissonance than no labels (M = 3.79, SD = 1.39; *p* < 0.001, Cohen’s *d* = 0.27, small effect), and ambiguous labels (M = 5.44, SD = 1.48) produced significantly higher dissonance than both no-label and clear-label conditions (both *p*-values <0.001). This suggests that ambiguous labels are most effective at eliciting psychological discomfort and imbalance, clear labels exert a moderate effect, and no labels elicit the lowest level of dissonance.

The main effect of label–content congruence was significant, *F*(1, 373) = 10.30, *p* = 0.001, $\eta_{p}^{2}$ = 0.027 (small effect), with higher cognitive dissonance in the inconsistency condition (M = 4.70, SD = 1.67) than in the consistency condition (M = 4.21, SD = 1.44). The main effect of AI thematic relevance was marginally significant, *F*(1, 373) = 3.13, *p* = 0.078, $\eta_{p}^{2}$ = 0.008, indicating a trend for implicit AI topics to elicit greater dissonance than explicit topics, although the effect size was small.

Regarding interactions, there was a significant interaction between AI label type and label–content congruence, *F*(2, 373) = 6.14, *p* = 0.002, $\eta_{p}^{2}$ = 0.032 (small effect), and a significant interaction between AI label type and AI thematic relevance, *F*(2, 373) = 3.90, *p* = 0.021, $\eta_{p}^{2}$ = 0.020 (small effect). The three-way interaction was not significant (*p* = 0.103). Simple effects analyses further revealed that the impact of AI label type on cognitive dissonance was amplified under label–content inconsistency and implicit AI topic conditions. That is, in more complex and ambiguous contexts, ambiguous or contradictory AI labels more strongly elicited cognitive dissonance. None of the covariates had significant effects in this model (all *p*-values >0.077).

#### 5.2.4 Mediation analysis

**Figure 6:**
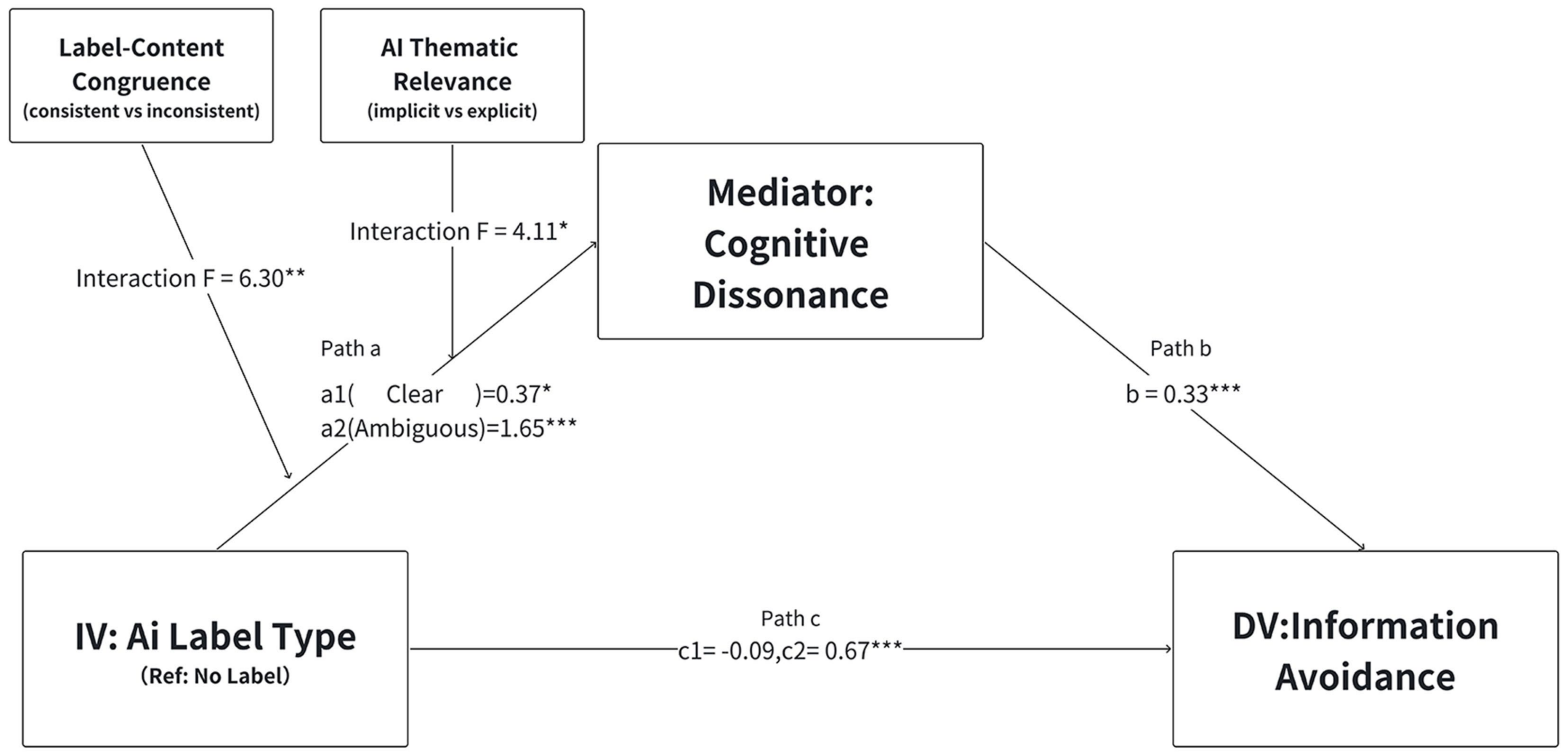
Results of the moderated mediation analysis for Study 2. Source: Author’s own work. Unstandardized coefficients are reported. Path coefficients (*b*) for the direct and indirect effects are derived from the simple mediation analysis (Model 4) to illustrate the overall directionality, while the *F* indicate the significance of interaction effects derived from the moderated mediation analysis (Model 9). *a*1 represents the effect of clear label vs. no label; *a*2 represents the effect of ambiguous label vs. no label. ^*^p < 0.05, ^**^p < 0.01, and ^***^p < 0.001.

Using PROCESS Model 4, we examined cognitive dissonance as a mediator linking AI label type to information avoidance, controlling for gender, age, education level, and daily internet use. The results are summarized in Table 4 and visualized in Figure 6. The regression model for the mediator (cognitive dissonance) was significant [*F*(6, 382) = 16.43, *p* < 0.001], explaining 20.5% of the variance (*R*^2^ = 0.205). Consistent with H2, cognitive dissonance positively predicted information avoidance (*b* = 0.33, *p* < 0.001). The overall model predicting avoidance was also significant (*R*^2^ = 0.257, *p* < 0.001) regarding the path from labels to dissonance, while clear labels increased dissonance (*b* = 0.37, *p* < 0.05), ambiguous labels triggered a substantially stronger response (*b* = 1.65, *p* < 0.001). Consequently, the indirect effect of ambiguous labels on avoidance (*b* = 0.54) was more than four times stronger than that of clear labels (*b* = 0.12). Regarding direct effects, ambiguous labels retained a significant impact (*b* = 0.67, *p* < 0.001), explaining significant additional variance (Δ*R*^2^ = 0.045), whereas clear labels did not (*p* = 0.557). Among covariates, only daily internet use reached significance (*p* = 0.036).

**Table 4:** Summary of simple (Model 4) and moderated (Model 9) mediation analyses.

Path/Condition	Effect (*b*)	SE	95% CI
Panel A: Simple mediation (Model 4)			
Independent variable: Clear label			
Direct effect	−0.09	0.16	[−0.41, 0.22]
Indirect effect via dissonance	0.12	0.06	[0.01, 0.24]
Independent variable: Ambiguous label			
Direct effect	0.67	0.18	[0.33, 1.02]
Indirect effect via dissonance	0.54	0.10	[0.35, 0.74]
Panel B: Conditional indirect effects (Model 9)			
Path: Ambiguous label avoidance			
Consistent + explicit AI	0.18	0.11	[−0.04, 0.41]
Consistent + implicit AI	0.46	0.12	[0.25, 0.72]
Inconsistent + explicit AI	0.57	0.13	[0.34, 0.83]
Inconsistent + implicit AI	0.86	0.16	[0.56, 1.18]
Indices of moderated mediation			
Moderator: Congruence	0.39	0.13	[0.17, 0.66]
Moderator: Relevance	0.29	0.12	[0.07, 0.55]

Taken together, these results indicate that cognitive dissonance significantly mediates the relationship between AI label type and information avoidance, particularly in the ambiguous-label condition, where labels increase dissonance and in turn enhance information avoidance.

#### 5.2.5 Moderated mediation analysis

To further examine the moderating roles of label–content congruence and AI thematic relevance on the path from AI label type to cognitive dissonance, Study 2 employed PROCESS Model 9, entering these two variables as dual moderators of the path from the independent variable to the mediator, while controlling for gender, age, education level, and daily internet use.

In the model with cognitive dissonance as the dependent variable, the overall model was significant, *R*^2^ = 0.274, *F*(12, 376) = 11.82, *p* < 0.001. Introducing the interaction terms involving label–content congruence significantly increased explained variance, Δ*R*^2^ = 0.024, *F*(2, 376) = 6.30, *p* = 0.002. Adding the interaction terms involving AI thematic relevance also significantly increased explained variance, Δ*R*^2^ = 0.016, *F*(2, 376) = 4.11, *p* = 0.017. Together, the two sets of moderators yielded a combined incremental explanatory power of Δ*R*^2^ = 0.041, *F*(4, 376) = 5.32, *p* < 0.001. Conditional effect analyses showed that the impact of AI label type on cognitive dissonance was more pronounced under label–content inconsistency and implicit AI topic conditions, suggesting that complex and ambiguous contexts considerably amplify label-induced dissonance.

In the model with information avoidance as the dependent variable, the overall model was identical to that in the previous mediation analysis, *R*^2^ = 0.257, *F*(7, 381) = 18.85, *p* < 0.001, with cognitive dissonance again positively predicting information avoidance (*b* = 0.325, *t* = 7.09, *p* < 0.001).

Table 4 (Panel B) presents the conditional indirect effects and indices of moderated mediation. The analysis revealed distinct boundary conditions for the two label types. For clear labels, the indirect path via cognitive dissonance was largely non-significant, emerging only under the specific condition where labels were inconsistent and the topic was implicit (*b* = 0.34). In contrast, for ambiguous labels, the indirect effect was robust and significant across most experimental conditions. Specifically, the avoidance effect peaked when an ambiguous label appeared on inconsistent content with an implicit theme (*b* = 0.86). The indices of moderated mediation further confirmed that both label–content congruence and AI thematic relevance significantly moderated the pathway for ambiguous labels, whereas only thematic relevance served as a significant moderator for clear labels.

### 5.3 Discussion

The results of Study 2 replicated and extended the core effects observed in Study 1, supporting H3 and further confirming H4 and H5. Consistent with the perspective that uncertainty amplifies defensive information avoidance (Dai et al., 2020), and that discomfort intensifies under ambiguous conditions (Metzger et al., 2020; Song et al., 2021), these findings highlight that, under inconsistent or implicit conditions, ambiguous AI labels more strongly drive avoidance through the cognitive dissonance pathway. Building on this, the general discussion integrates the results from both studies and elaborates their theoretical and practical implications.

## 6 Overall discussion

### 6.1 Summary of the findings

Across two experiments with a combined sample of 760 participants, this research shows that AI disclosure labels systematically shape users’ information avoidance in short-video environments. Ambiguous warnings that merely suggest possible AI involvement elicit the highest levels of avoidance compared with both clear labels and the absence of labels, and this pattern replicates from a controlled Bilibili-style setting to a more ecological TikTok-style context. Clear labels, in contrast, do not consistently increase avoidance relative to no label, which suggests that ambiguity rather than transparency is the primary driver of defensive disengagement.

Cognitive dissonance emerges as a central psychological mechanism behind this effect. Higher perceived dissonance robustly predicts more avoidance in both studies, and mediation analyses show that label type influences avoidance largely through dissonance, with ambiguous labels having the greatest indirect effect.

In Study 2, these processes are context dependent. When the platform labels are incongruent with the actual video generation process or AI is only implicitly related to the topic, dissonance and downstream avoidance increase, especially for ambiguous labels. However, for congruent labels in explicitly AI-themed content, these reactions decrease.

Collectively, the findings indicate that ambiguous AI disclosures function as salient uncertainty cues that deter engagement, while clear labels mainly shape behavior through dissonance in situations where transparency conflicts with users’ prior expectations. Reduced engagement in labeled content, particularly under ambiguous conditions, reflects users’ strategic disengagement from epistemic ambiguity, as evidenced by elevated avoidance scores in ambiguous groups (Study 1: M = 4.05 vs. no label M = 3.37; Study 2: M = 4.45 vs. no label M = 3.23). The data demonstrates that label-content mismatches and implicit AI themes amplify this avoidance behavior (Cohen’s *d* = 0.32 to 0.57 in mismatch scenarios). These patterns suggest that ambiguous labels function not merely as metadata, but as heuristic barriers that fundamentally alter user navigation flows.

Mechanistically, cognitive dissonance links label type and avoidance via a psychological pathway in which ambiguity disrupts cognitive coherence and is resolved with minimal effort by disengaging. Ambiguous labels challenge user expectations by introducing unresolved uncertainty, reducing processing fluency and highlighting authorship conflicts, as supported by indirect effects (Study 1: *b* = 0.44; Study 2: *b* = 0.537) that account for a significant proportion of the variance in avoidance. This highlights dissonance as an affective driver. Moderated mediation shows that incongruence and implicit themes strengthen this path (index *b* = 0.394 to 0.285), explaining why vague cues increase processing demands in overloaded feeds, thus prioritizing cognitive economy over content evaluation and extending action-based dissonance models to algorithmic contexts.

### 6.2 Theoretical implications

#### 6.2.1 AI disclosures and defensive disengagement

The findings refine theorizing on AI disclosure labels in digital media by challenging existing frameworks that conceptualize labels primarily as transparency tools for identifying synthetic media and calibrating trust, assuming they promote scrutiny rather than disengagement (Li et al., 2023; Wittenberg et al., 2024, 2025). Recent design oriented work shifts attention to specific warning formats and examines how variations in label sentiment, iconography, placement, and detail influence perceived authenticity, label credibility, and platform trust, while generally finding only modest changes in likes, comments, and sharing (Gamage et al., 2025). The present results extend this body of research by demonstrating that in high-velocity short-video feeds, ambiguous AI labels operate not merely as peripheral cues but as upstream validity filters. While content relevance remains a central driver of engagement (Berger and Milkman, 2012), our data implies that ambiguous labels introduce a layer of epistemic risk that can impede this process. In this context, vague disclosures function as heuristic barriers that push users toward defensive disengagement before the thematic value of the content is fully processed. Unlike clear labels which may act as neutral metadata, ambiguous labels effectively impede the engagement process by signaling unresolved epistemic risk, thus outweighing potential topical interest in the initial filtering stage.

#### 6.2.2 Cognitive dissonance in algorithmic environments

Furthermore, it adds to cognitive dissonance and info-avoidance theories in an algorithmically curated setting, building off of classic selective-exposure research which frames avoidance as a way to manage dissonance due to incongruent information (Hart et al., 2009). Related economic psychology research has found that people may strategically avoid noisy, stochastic, or fake information even if its revelation is objectively good for them (Momsen and Ohndorf, 2022). The moderated mediation model tested here is nuanced as it shows that in the context of AIGC, dissonance can be triggered by meta-informational cues about authorship and reliability rather than ideological conflict. We characterize this state as epistemic dissonance, defined in this context as the specific conflict between the user’s truth-default expectation (Levine, 2014) and the platform’s uncertainty warning. This state is aversive not just because of the cognitive effort required to verify, but because it disrupts the cognitive fluency needed for hedonic consumption. People thus often choose to skip the labeled content entirely. This extends action-based accounts of dissonance by foregrounding avoidance of algorithmically queued items as a central behavioral route through which users restore psychological consistency on social media.

#### 6.2.3 Labels as informational and affective cues

Finally, these findings reconceptualize AI disclosure labels beyond their traditional role as passive transparency metadata (Epstein et al., 2023; Wittenberg et al., 2025). Instead, our results suggest they function as active informational interventions and validity gatekeepers that elicit affective responses which fundamentally alter user processing. Unlike neutral content tags, ambiguous AI labels trigger rapid avoidance responses by inducing cognitive dissonance, effectively reallocating user attention away from content evaluation toward dissonance reduction. This perspective complements recent HCI work showing that label designs shift user belief without necessarily reducing engagement (Gallegos et al., 2025; Gamage et al., 2025). However, we extend this by demonstrating that ambiguous labels act as preemptive disruptors that disrupt the cognitive flow in high-velocity media environments. By intercepting the user journey at the validity assessment stage, these labels can prompt users to abort the subsequent content consumption goal, rather than merely serving as helpful accountability signals.

### 6.3 Practical implications

#### 6.3.1 Interface design and ambiguity reduction

Platform designers can transition from suspicion-based warnings to definitive presentation strategies based on high-confidence detection. Our findings demonstrate that ambiguous labels function as cognitive barriers rather than helpful signals, triggering the highest levels of information avoidance compared to clear disclosures. This avoidance stems from cognitive dissonance, where the uncertainty of a Suspected AI tag forces users to expend mental effort on verification during leisure consumption (Festinger, 1957; Golman et al., 2017). To mitigate this, platforms should minimize the exposure of probabilistic indicators that effectively offload algorithmic uncertainty onto users (Ananny and Crawford, 2018). Instead, design protocols should prioritize high-threshold binary classification, displaying labels only when the system reaches a sufficient confidence level to warrant a definitive assertion. Furthermore, the visual presentation of these labels must ensure immediate comprehension without requiring secondary interactions. Since increased processing costs drive disengagement (Kahneman, 2011), transparency cues should be self-explanatory and glanceable to maintain user flow while ensuring informed consumption.

#### 6.3.2 Provenance standards and policy shifts

Regulatory frameworks must evolve to prioritize information certainty over broad disclosure mandates. Our results indicate that wide-ranging warnings derived from probabilistic AI detection tools inadvertently disincentivize content consumption. Specifically, the data shows that ambiguous labels, which often result from uncertain algorithmic detection (Sadasivan et al., 2023), trigger a defensive avoidance response that is significantly stronger than that of clear labels. This suggests that policies encouraging platforms to flag potential AI content based on estimation create a user experience characterized by cognitive dissonance rather than transparency. Consequently, policy governance should shift focus from mandating detection-based warnings to supporting provenance-based authentication standards. The primary advantage of provenance mechanisms in this context is their ability to provide more definitive attribution compared to probabilistic detection. By anchoring disclosure in verified source data, regulators can facilitate the linguistic clarity necessary for users to evaluate content without the psychological burden of uncertainty. This approach aligns transparency goals with the cognitive needs of users, preventing the unnecessary disengagement caused by vague suspicion markers.

#### 6.3.3 Proactive disclosure and creator strategies

Content creators and advertisers should view proactive disclosure as a strategic defense mechanism rather than merely an ethical obligation. The experimental results demonstrate that label incongruence significantly amplifies user avoidance. Specifically, the data reveals that when a label contradicts the actual nature of the content or appears unexpectedly, information avoidance scores rise markedly. This indicates that leaving disclosure to platform algorithms carries a high risk. If an automated system incorrectly tags human content or subtly edited media with a vague suspicion marker, the creator suffers from the ambiguity penalty identified in this study. By voluntarily applying clear and accurate labels, creators effectively mitigate the assignment of high-friction ambiguous tags. This strategy maintains control over the reception of the content and facilitates that the audience processes the material with the intended fluency (Reber et al., 2004), avoiding the cognitive dissonance that drives users to scroll past uncertain stimuli.

### 6.4 Limitations and future research

Several limitations of this study should be acknowledged when interpreting the findings and they open promising directions for future work. First, the experiments relied on short form video scenarios that mimicked Bilibili and TikTok feeds with a specific set of AI label wordings and visual styles. This design improves internal validity but constrains generalizability to other platforms and labeling schemes including emerging provenance style badges and automatic metadata based labels now adopted by TikTok and YouTube (TikTok, 2024). This underscores methodological concerns about ecological validity in social media research, as simulated feeds may not fully capture dynamic, algorithm-driven, multitasking contexts and self-reported intentions often diverge from actual in-situ scrolling behaviors due to social desirability or recall errors (Griffioen et al., 2020; Parry et al., 2022). Future research could test the proposed dissonance based mechanism in more diverse interfaces such as news websites, streaming platforms or messaging apps using objective log-level data to validate these effects.

Second, the study’s sample characteristics may limit the universality of the findings. The reliance on Chinese users and short-term exposure scenarios potentially overlooks variations driven by cultural context and individual traits. Cross-national surveys suggest that comfort with AI-authored content varies significantly across countries (Fletcher and Nielsen, 2024), and individual differences in AI literacy or need for cognition could further moderate avoidance thresholds (Ding et al., 2025). Future work should adopt cross-cultural and stratified designs to examine how these user characteristics influence label susceptibility building on studies of accuracy perception (Altay and Gilardi, 2024).

Third, the focus on cognitive dissonance as the central mechanism may not fully account for the competing influence of content utility under extreme conditions. While our validity gatekeeper framework suggests that ambiguous labels can effectively inhibit engagement, significant boundary conditions likely exist. In naturalistic settings, extremely high intrinsic user interest or informational necessity (e.g., urgent health crises or exclusive fandom content) could arguably override the heuristic barrier of the label. This represents a boundary condition that this controlled experiment did not empirically identify (Golman et al., 2017). Furthermore, the current framework prioritizes dissonance over factors such as credibility judgments, information overload, and identity-motivated selective exposure (Harmon-Jones, 2019). Future studies could test multi-process models to identify the specific utility threshold where content relevance supersedes the inhibitory effect of transparency cues (Moravec et al., 2019), while contrasting dissonance with alternatives like defensive pessimism.

Finally, the study examined static labels in low-stakes entertainment contexts, leaving the dynamics of sensitive topics and long-term adaptation unexplored. In high-risk domains such as elections or public health, users may demand greater transparency (Li et al., 2023; Piasecki et al., 2024; Wittenberg et al., 2025), yet they may also adapt to repeated exposures as generative AI routinizes media production. Future research should explore ambiguous labels in sensitive topics paired with media literacy interventions (Himmelroos et al., 2024) and conduct longitudinal studies to examine whether labels eventually become noise or if avoidance patterns shift as users’ notions of authenticity evolve (Lewis et al., 2025).

## 7 Conclusion

This study reveals a critical paradox in AI content labeling on social media platforms: while intended to enhance transparency and user scrutiny, ambiguous AI disclosures inadvertently heighten information avoidance through cognitive dissonance, particularly in mismatched or implicitly themed contexts. Across two experiments (*N* = 760), ambiguous labels consistently drove the strongest avoidance effects, mediated by dissonance and moderated by contextual factors, challenging assumptions that all transparency cues uniformly promote engagement. These insights extend cognitive dissonance and information avoidance theories to AI-augmented ecosystems, emphasizing the need for precise, clear labeling strategies that minimize heuristic barriers to engagement. For platforms, regulators, and creators, prioritizing provenance-oriented disclosures over vague warnings can reduce epistemic uncertainty, while minimizing unnecessary friction, ultimately supporting more accountable and cognitively aligned digital environments. Future work should explore cross-cultural adaptations and longitudinal impacts to refine these interventions in evolving media landscapes.

## Data Availability

The raw data supporting the conclusions of this article will be made available by the authors, without undue reservation.
